# Changes in Hepatic Blood Flow and Liver Function during Closed Abdominal Hyperthermic Intraperitoneal Chemotherapy following Cytoreduction Surgery

**DOI:** 10.1155/2018/8063097

**Published:** 2018-03-12

**Authors:** Stéphanie Dupont, Eduardo R. C. Schiffer, Marion J. White, John R. A. Diaper, Marc-Joseph Licker, Philippe C. Masouyé

**Affiliations:** Geneva University Hospitals, Geneva, Switzerland

## Abstract

**Background:**

The increase in intra-abdominal pressure (IAP) during closed abdominal hyperthermic intraperitoneal chemotherapy (HIPEC) leads to major haemodynamic changes and potential organ dysfunction. We investigated these effects on hepatic blood flow (HBF) and liver function in patients undergoing HIPEC following cytoreductive surgery and fluid management guided by dynamic preload indices.

**Methods:**

In this prospective observational clinical study including 15 consecutive patients, we evaluated HBF by transesophageal echocardiography and liver function by determination of the indocyanine green plasma disappearance rate (ICG-PDR). Friedman's two-way analysis of variance by ranks and Wilcoxon signed-rank test were performed for statistical analysis.

**Results:**

During HIPEC, HBF was markedly reduced, resulting in the loss of any pulsatile Doppler flow signal in all but one patient. The ICG-PDR, expressed as median (interquartile 25–75), decreased from 23 (20–30) %/min to 18 (12.5–19) %/min (*p* < 0.001). Despite a generous crystalloid infusion rate (27 (22–35) ml/kg/h), cardiac index decreased during the increased IAP period, inferior vena cava diameter decreased, stroke volume variation and pulse pressure variation increased, lung compliance dropped, and there was an augmentation in plateau pressure. All changes were significant (*p* < 0.001) and reversed to baseline values post HIPEC.

**Conclusion:**

Despite optimizing intravenous fluids during closed abdominal HIPEC, we observed a marked decrease in HBF and liver function. Both effects were transient and limited to the period of HIPEC but could influence the choice between closed or open abdominal cavity procedure for HIPEC and should be considered in similar clinical situations of increased IAP.

## 1. Introduction

Cytoreductive surgery (CRS) with hyperthermic intraperitoneal chemotherapy (HIPEC) is an emerging treatment for peritoneal carcinomatosis [1, 2]. This major cytoreductive abdominal surgery is followed by intraperitoneal filling with a heated chemotherapy solution [3] either with an open abdominal technique (Coliseum technique) [4] or closed abdominal technique. This second procedure provokes an increase in intra-abdominal pressure (IAP) associated to a HIPEC-induced hyperdynamic circulatory state heat stress response, both leading to major haemodynamic changes [5]. Changes in hepatic blood flow (HBF) have been reported in adults undergoing laparoscopic surgery [6, 7] most probably related to the IAP required for this procedure. In addition, intra-abdominal hypertension (IAH) or acute abdominal compartment syndrome (ACS) can decrease hepatosplanchnic flow and reduce liver function [8, 9]. The aim of the present study was to investigate the effects of increased IAP during HIPEC on HBF and liver function.

## 2. Methods

This was a single center prospective cohort observational study.

Following approval of our institutional ethics committee (Commission centrale d'éthique de la recherche sur l'être humain, CER: 10-193, NAC 10-066) and informed written consent, 15 adult patients with peritoneal carcinomatosis scheduled for elective CRS with HIPEC were consecutively enrolled between March 2011 and March 2015 at our university hospital single surgical center. Patients were selected unrelated to the origin of the primary neoplasm.

Exclusion criteria before surgery were contraindications to transesophageal echocardiography (TEE) (esophageal or gastric lesions), atrial fibrillation, or incapacity to give informed consent. Patients allergic to indocyanine green (ICG) or iodine, or with thyrotoxicosis, were not investigated with the ICG clearance method.

### 2.1. Anaesthesia and Surgical Management

Standard patient monitoring was supplemented by a radial artery catheter that was connected to the LiDCO haemodynamic monitor (LiDCO Ltd., London, UK). A thoracic epidural catheter was placed for pre- and postoperative analgesia (bupivacaïne 0.25%, 6–10 ml/h). General anaesthesia was induced with intravenous bolus of sufentanil (10 mcg) and propofol (1.5–2.5 mg/kg) and maintained with desflurane [10] to obtain a Bispectral Index value between 40 and 50. Neuromuscular block was achieved with atracurium 0.5 mg/kg or suxamethonium 1 mg/kg for orotracheal intubation. Patients were ventilated with a 40% mixture of O_2_/air, a tidal volume of 5–7 ml/kg, a respiratory rate of 10–14/min, and a positive end-expiratory pressure of 5 cm H_2_O.

Nasopharyngeal and rectal temperature probe readings were recorded. Transesophageal echocardiography (Philips X7-2t, Bothell, WA, USA) allowed for hepatic echo and Doppler measures. Antibiotic prophylaxis was administered and antiembolic leg cuffs were applied. Fluid bolus challenges were given at regular intervals during surgery and assessed using the dynamic response screen on the LiDCO monitor. Cardiac index, stroke volume index, stroke volume variation (SVV), and pulse pressure variation (PPV) were all maintained at normal limits prior to HIPEC [11].

Body temperature was maintained above 35.5°C during the CRS with a pulsed air warming blanket, a warming perfusion device, and a thermal water mattress. At the end of CRS, the patient was actively cooled by an intraperitoneal cold solution irrigation, cold intravenous infusions, and the cooling water mattress to reach a body temperature of approximately 33°C. Abdominal cavity was closed and HIPEC procedure was started: intraperitoneal cavity was filled through abdominal drains until sufficient abdominal wall tension appreciated by the surgeon was obtained; then, once the extracorporeal circuit full flow was achieved, the heated chemotherapy solution was infused.

### 2.2. Measurement of HBF and Liver Function

Right and middle vein HBF was measured with transesophageal echocardiography Doppler using the method previously described by Meierhenrich [12] and the following formula:

(1) $$\begin{array}{c} Blood\;flow\;\left(ml/\min\right)=k\times VTI\;\left(cm\right)\times \pi \;r^{2}\;\left({cm}^{2}\right)\times HR\;\left(beats/\min\right), \end{array}$$

where VTI = velocity time integral, *πr*^2^ = cross-sectional area of the vessel, HR = heart rate, and *k* = 0.7. Blood flow measures were indexed to the body surface area. Right and middle hepatic vein diameters and Doppler signals, inferior vena cava (IVC) diameter, and left ventricle ejection fraction (LVEF) obtained by Simpson method or 3D quantification were measured at end-expiration during three cardiac cycles and averaged. All transesophageal echocardiography measurements were obtained by the same nonblinded observer (John R. A. Diaper), and the data were analyzed using the QLab program on the Philips iE33 Ultrasound Machine (Koninklijke Philips N.V.). All transesophageal echocardiography data were analyzed and supervised online by the Head of cardiovascular anesthesiology and specialist in transesophageal echocardiography (Marc-Joseph Licker).

Liver function was measured by ICG clearance (LiMON liver function monitor, PULSION Medical Systems AG, Munich, Germany) [13, 14]. ICG removal from the blood depends on liver blood flow, parenchymal cellular function, and biliary excretion. After an intravenous bolus of 0.5 mg/kg ICG (as sodium iodine), ICG elimination was expressed as indocyanine green plasma disappearance rate (ICG-PDR). For ICG-PDR, initial concentration at time 0 is normalized to 100% and ICG-PDR is the percentage change over time (percent per minute). ICG-PDR was measured noninvasively by finger probe.

### 2.3. Measurement of IAP

IAP was estimated from the pressure in the extracorporeal inlet line at abdominal insertion and secondly at full flow (4-5 l/min). Delta *P* was the difference between these two measures.

### 2.4. HIPEC Protocols

The duration of HIPEC was 30 min or 90 min depending on the neoplasm origin with either leucovorin, 5-fluorouracil, mitomycin, or oxaliplatin for chemotherapy.

### 2.5. Perioperative Time Measurements

- Time 0: after induction of general anaesthesia, before starting epidural analgesia
- Time 1: 30 min after beginning epidural analgesia
- Time 2: end of CRS, before abdominal filling
- Time 3: end of HIPEC, before abdominal emptying
- Time 4: end of procedure, after abdominal emptying and removal of abdominal drainage

Biological serum parameters (aspartate aminotransferase (ASAT), alanine aminotransferase (ALAT), alkaline phosphatase, bilirubin, gamma-glutamyltranspeptidase, protein, albumin, urea, creatinine, prothrombin time, activated partial thromboplastin time, and fibrinogen) and complete blood count were recorded on the preoperative day, the operative day on arrival in the Intensive Care Unit (ICU), and for the next three days.

### 2.6. Statistics

Sample size was determined to detect a 40% decrease in HBF with a power of >80% and a type 1 error probability of 5%. Fifteen patients provided the necessary power allowing for dropouts and study withdrawals. All data are presented as median and (quartile 25–quartile 75) range. Friedman's two-way analysis of variance by ranks was applied for multiple comparisons over time. Wilcoxon signed-rank test was performed to compare parameters between two time points. A two-tailed *P* value < 0.05 was considered statistically significant. Statistics and graphs were performed using SPSS software release 15 (2009 SPSS^©^ Inc. Headquarters, 11th floor, 233 S. Wacker Drive, Chicago, Illinois 60606).

#### 2.6.1. Primary Endpoints

Transesophageal echocardiography measured HBF and liver function by determination of the ICG-PDR.

#### 2.6.2. Secondary Endpoints

Biological, haemodynamic, respiratory, and general parameters (amount of fluids infused, intraoperative staging of the peritoneal carcinomatosis, parameters related to epidural analgesia, need of inotrope/vasopressor support, duration of the CRS, and circuit pressure) were analyzed.

## 3. Results

Fifteen patients (10 females) scored ASA 2 were consecutively included in our study. No patient was excluded. Median age was 54 (48–63) years, body weight 70 (51–76) kg, and height 165 (157–178) cm. The origin of carcinoma was sigmoid (4), caecum (1), right colon (1), appendix (4), ovarian (2), gastric (2), and mesothelium (1). Peritoneal carcinomatosis index (PCI) was 6 (0–20) and CRS time was 300 min (240–330). A 30 min HIPEC procedure was applied for 10 patients and a 90 min HIPEC procedure for 5 patients without any difference of hepatic or haemodynamic changes between these 2 groups. Abdominal cavity filling was 2.38 (1.85–3.12) l/m^2^. Once abdominal filling was reached to allow full flow on the circuit, intra-abdominal pressure stayed constant during both 30 min and 90 min HIPEC procedures. Patients undergoing either 30 min or 90 min HIPEC protocols showed similar changes in ICG-PDR values or hepatic blood flow. During the HIPEC procedure, circuit pressure was 30 mmHg (28–43) at abdominal insertion of the inlet line and raised to 62 mmHg (43–70) at full flow (delta *P* = 19 mmHg (15–40)).

**Figure 1:**
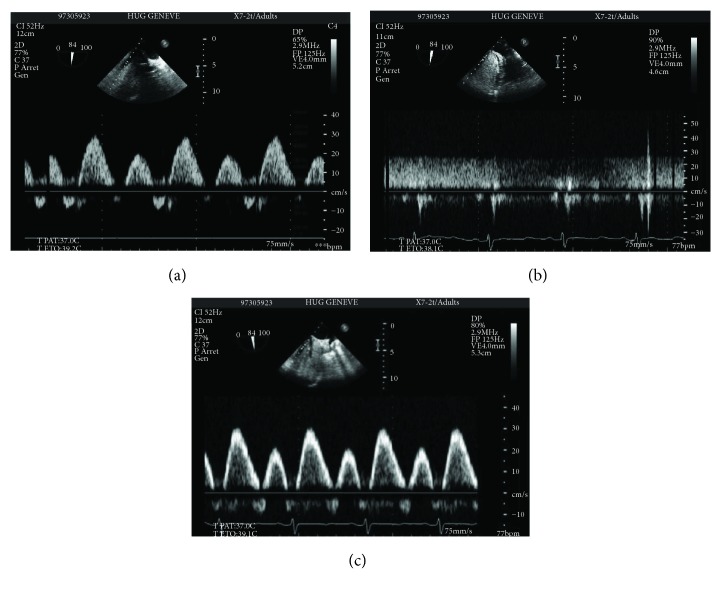
Hepatic blood flow at the middle hepatic vein measured by transesophageal echocardiography. (a) Normal triphasic Doppler signal of HBF from the MHV at time 2 (end of CRS, before abdominal filling). (b) Nonpulsatile Doppler signal at time 3 (end of HIPEC, before abdominal emptying). (c) After emptying abdominal cavity, at time 4 (end of procedure, after abdominal emptying and removal of abdominal drainage), HBF returns to normal Doppler signal waveform. HBF: hepatic blood flow; MHV: middle hepatic vein.

**Figure 2:**
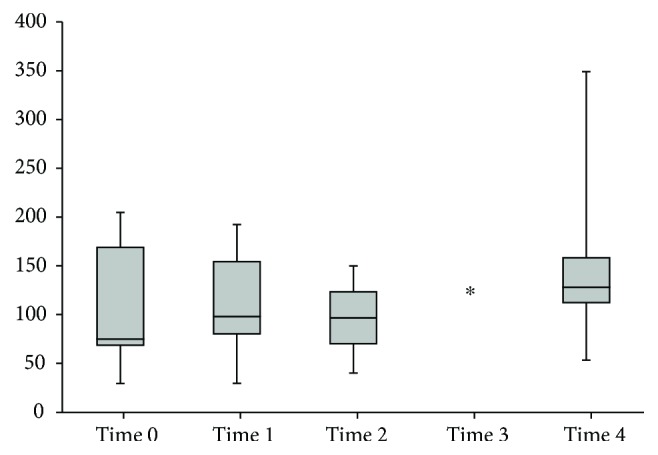
Middle hepatic vein blood flow index measured by transesophageal echocardiography. Box and whisker diagram of middle hepatic vein blood flow index expressed in ml/min/m^2^. Whisker extremities are maximum and minimum, boxes are interquartile ranges between the 75th and 25th percentiles, and the segment inside the box is the median of the sample. *P* value < 0.001 results from a Friedman's two-way analysis of variance by rank. The ∗ indicates a significantly different value compared to baseline with the Wilcoxon signed-rank test. Time 0: after induction of general anaesthesia, before starting epidural analgesia. Time 1: 30 min after beginning epidural analgesia. Time 2: end of CRS, before abdominal filling. Time 3: end of HIPEC, before abdominal emptying. Time 4: end of procedure, after abdominal emptying and removal of abdominal drainage.

**Figure 3:**
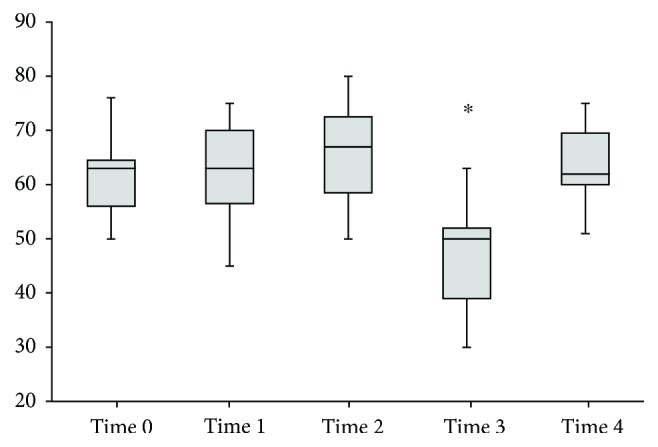
Left ventricle ejection fraction measured by transesophageal echocardiography. Box and whisker diagram of left ventricle ejection fraction expressed in %. Whisker extremities are maximum and minimum, boxes are interquartile ranges between the 75th and 25th percentiles, and the segment inside the box is the median of the sample. *P* value < 0.001 results from a Friedman's two-way analysis of variance by rank. The ∗ indicates a significantly different value compared to baseline with the Wilcoxon signed-rank test. Time 0: after induction of general anaesthesia, before starting epidural analgesia. Time 1: 30 min after beginning epidural analgesia. Time 2: end of CRS, before abdominal filling. Time 3: end of HIPEC, before abdominal emptying. Time 4: end of procedure, after abdominal emptying and removal of abdominal drainage.

Intraoperative changes in hepatic and systemic haemodynamic variables are presented in Table 1. During HIPEC, HBF decreased sharply (Figures 1 and 2, Table 1) and Doppler signals became nonpulsatile in the right and middle hepatic veins, except for one patient in whom pulsatile signals were maintained despite a major decrease in HBF (70–80%). ICG-PDR significantly decreased from 23 (20–30) to 18 (8.3–30) %/min, reflecting a lower liver extraction, and returned to baseline at time 4. Liver enzymes and serum lactates significantly increased with the procedure and decreased progressively in the postoperative days (Table 2). From the beginning of the intraperitoneal filling, during HIPEC and up to the end of surgery, a significant but moderate increase in heart rate was observed. Mean arterial pressure remained stable throughout the procedure. Cardiac index increased significantly after initiation of epidural analgesia returning to similar baseline values at time 3. At time 4, cardiac index reincreased significantly compared to baseline and to time 3 value. During HIPEC, LVEF significantly decreased (Figure 3), and measurements of intravascular filling (SVV, PPV, and IVC diameter) suggested significant hypovolaemia.

There was no decrease in diuresis or increase in serum creatinine during HIPEC and the first postoperative days. Loop diuretic or mannitol were not administered perioperatively. Crystalloid infusion was 8300 (6000–13,500) ml of Ringer's acetate solution, representing 27 (22–35) ml/kg/H. Six patients received colloids (Voluven^®^) and 4 patients were transfused. Continuous norepinephrine (100 to 200 *μ*g/H) was administered in 8 patients to maintain haemodynamics.

At time 3, statistically significant respiratory changes included decreased lung compliance from 48.5 (37–52) to 27 (16.3–30) ml/cm H_2_O, increased plateau pressure from 16 (13–21) to 27 (25–29) cm H_2_O, and decreased oxygenation index (PaO_2_/FiO_2_). All respiratory parameters improved at time 4 (Table 1).

No patient suffered from potential complications related to epidural technique (spinal hematoma, meningitis, abscesses, and haemodynamic disorders), or bleeding disorders, such as delusional coagulopathy. Coagulation profile was stable except for a postoperative increase in serum fibrinogen probably related to an inflammatory state.

Intensive Care Unit (ICU) stay was 2 (2, 3) days. All patients were discharged home within 10–15 days. One patient died 21 months after surgery, and all others survived.

## 4. Discussion

Since the early nineties, the effects of increased-IAP have been described in animals [15] and humans [16] especially in relation to the development of abdominal laparoscopic techniques, which are now a reference for many digestive, urological, and gynaecological surgical procedures. The effects of IAP during laparoscopy on the splanchnic circulation have been recently reviewed [17], and the impact of increased IAP has been the subject of renewed interest in ICU during organ failure associated with acute ACS [18]. Deleterious haemodynamic effects [19] and repercussions on HBF [20] appear in most of the studies with a threshold limit of approximately 14 mmHg [21]. Although we did not measure the IAP directly, our study shows that increased IAP during closed abdominal HIPEC procedure leads to an important decrease in HBF, associated with a transient alteration in liver function, increased serum transaminases, and lactates most probably related to acute hepatic ischemia. This effect was reversible in less than 48 hours and had no impact on the clinical outcome. The compensatory mechanism known as hepatic artery buffer response (HABR) which leads to an increase in the hepatic arterial blood flow in circumstances where the portal flow is reduced, allowing by that a partially maintained hepatic blood flow and subsequently adequate hepatic clearance and oxygen supply [22], could state for the relative good tolerance by the liver of decreased hepatic blood flow induced by the closed abdominal procedure for HIPEC.

It could also be suggested that the closed abdominal technique, by reducing venous HBF and thus chemotherapy exposure, protects the liver, which could influence the choice between closed or open abdominal cavity procedure for HIPEC.

In half of the cases in our study, major reductions in HBF occurred for a moderate (<20 mmHg) pressure increase. Studies during laparoscopic cholecystectomy have shown a reduced HBF for low values of IAP (9–12 mmHg) [7] and a 100% increase in ALAT and ASAT 24 hours postoperatively in patients with decreased abdominal perfusion pressure (IAP values of 14 mmHg) [23]. Decreased liver function measured by ICG clearance has been associated with moderately increased IAP, suggestive of decreased splanchnic perfusion [24]. Minimally invasive techniques (transesophageal echocardiography or ICG clearance) could measure the effects of moderate increase in IAP and allow early detection of adverse effects in patients at risk of ACS [25]. Meierhenrich et al. [6] reported a 50% increase in HBF during the initial insufflation phase of laparoscopic surgery, possibly linked to an initial increase in venous return or to stimulation of the sympathetic and renin-angiotensin systems [26]. In contrast to these results, we did not witness similar increases in HBF at any time in our study. Although available for several years, CRS with HIPEC has long been considered experimental and was only recently validated as a treatment of peritoneal carcinomatosis [27]. This explains the increasing number of publications on the anaesthetic management of this procedure [28–33]. Considering it a high-risk surgery, with significant volaemic shifts, we chose a fluid management regime guided by dynamic preload indices (SVV, PPV) derived from the LiDCO monitor [34]. Total crystalloid requirement during the procedure [32] was comparable to standard fluid regimes for this operation [35]. Despite this optimized fluid management associated with vasopressors when required to maintain stable haemodynamics, increased IAP resulted in a significant decrease in IVC diameter indicating a reduced venous return and a major reduction in HBF. This resulted in a preload-dependent situation reflected by the concomitant increase in PPV and SVV, a decrease in cardiac index, and a significant reduction in LVEF. Further, venous return, in the context of increased IAP, is also influenced by decreased diaphragmatic dynamics, decreased lung compliance, and increased ventilatory inspiratory pressure. All these factors can contribute to gas exchange deterioration (decreased PaO_2_/FiO_2_) [36]. With abdominal cavity emptying, these changes were quickly reversible, except for the altered pH values and increased lactate serum levels which returned to normal in 2 days, as previously reported [31].

### 4.1. Study's Limitations

Our study is an observational study with a small case series. It meets appropriate settings for case series study design [37], reporting sentinel events (changes in HBF and liver function) induced by a novel treatment (HIPEC) of a nonfrequent disease (peritoneal carcinomatosis). Measurements of HBF by TEE have certain limitations as the left hepatic vein in not accessible in about 80% of anesthetized patients. In addition, the major source of error resides in the measurements of the cross-sectional area of the hepatic vein. Such difficulty in obtaining a precise measurement lies in the resolution of the ultrasound scanner, and the consequent absolute error in HBF is expected to be in the order of 16–20% [38]. In the current study, the laminar pattern of hepatic venous blood flow could easily be measured by TEE and it remains stable in all study periods, except after HIPEC induction (time 3). The sharp decline in HBF was explained by complete obstruction of the “collapsible” veins by the increased abdominal pressure.

## 5. Conclusion

Despite a fluid strategy guided by dynamic preload indices, increased IAP linked to closed abdominal HIPEC which resulted in a marked decrease of HBF, as measured by transesophageal echocardiography Doppler signals, and decreased liver function (ICG-PDR). These effects were transient and without clinical consequences but could influence the choice between closed or open abdominal cavity procedure for HIPEC and should be considered in clinical situations of increased IAP as in oncologic patients undergoing this treatment, in patients undergoing long abdominal laparoscopic surgery, especially obese patients requiring high IAP to maintain an optimal surgical vision, and in patients at risk of ACS.

## Figures and Tables

**Table 1:** Intraoperative changes in hepatic, systemic, respiratory, and blood gas parameters.

	Time 0	Time 1	Time 2	Time 3	Time 4	*P* value
RHVI blood flow (ml/min/m^2^)	172 (104–213)	214 (103–290)	215 (97–265)	Unmeasurable	197 (125–233)	<0.001
MHVI blood flow (ml/min/m^2^)	75 (68–172)	98 (77–172)	97 (68–134)	Unmeasurable	128 (110–164)	<0.001
ICG-PDR (%/min)	23 (20–30)	22 (20–24)	21 (19–24)	18^∗^ (13–19)	27 (24–39)	<0.001
Heart rate (beats/min)	64 (55–72)	72 (60–85)	79^∗^ (66–80)	82^∗^ (70–91)	80^∗^ (78–93)	<0.001
MAP (mmHg)	65 (59–92)	80 (70–88)	74 (70–83)	74 (69–80)	74 (66–88)	0.619
Cardiac index (ml/min/m^2^)	2.6 (2.2–2.9)	3.1^∗^ (2.4–3.5)	2.8 (2.4–3.1)	2.5 (1.9–3.0)	3.2^∗^ (2.9–3.8)	0.006
LVEF (%)	63 (52–65)	63 (56–70)	65 (57–73)	50^∗^ (38–52)	64 (60–70)	<0.001
SVV (%)	6 (4–8)	7 (5–13)	9^∗^ (7–11)	16^∗^ (10–21)	8 (6–10)	<0.001
PPV (%)	8 (5–10)	8 (7–15)	10 (9–13)	19^∗^ (12–27)	11 (8–15)	<0.001
IVC diameter (cm)	1.78 (1.37–2.04)	1.60 (1.39–1.95)	1.60 (1.40–1.98)	1.14^∗^ (0.90–1.30)	1.93 (1.67–2.12)	<0.001
Cumulated (diuresis ml)	30 (0–100)	300^∗^ (140–400)	450^∗^ (300–800)	550^∗^ (380–1100)	700^∗^ (460–1170)	<0.001
Compliance (ml/cm H_2_O)	48.5 (37–52)	42.0 (34–56)	48.5 (31–63)	27.0^∗^ (16–30)	34.5 (24–50)	<0.001
Plateau pressure (cm H_2_O)	16 (13–21)	17 (14–21)	15 (14–23)	27^∗^ (25–29)	20 (17–24)	<0.001
PaO_2_/FiO_2_ (mmHg)	373 (304–446)	310 (278–434)	450^∗^ (379–516)	280^†^ (196–405)	325 (228–399)	0.027
PaCO_2_ (mmHg)	36 (30–38)	31 (29–37)	34 (32–38)	32 (29–34)	36 (31–40)	0.264
pH	7.42 (7.40–7.48)	7.41 (7.38–7.46)	7.39 (7.37–7.43)	7.37^∗^ (7.32–7.40)	7.32^∗^ (7.29–7.35)	<0.001
Serum lactate (mmol/l)	0.95 (0.7–1.2)	0.90 (0.7–1.4)	1.70^∗^ (0.9–3.3)	1.90^∗^ (1.6–2.9)	2.80^∗^ (1.7–3)	<0.001

Data are expressed as median (quartile 25–quartile 75). *P* value results from a Friedman's two-way analysis of variance by rank. ∗ indicates a significantly different value compared to baseline and † indicates a significantly different value compared to time 2 value with the Wilcoxon signed-rank test. RHVI: right hepatic vein index; MHVI: middle hepatic vein index; ICG-PDR: indocyanine green plasma disappearance rate; MAP: mean arterial pressure; LVEF: left ventricle ejection fraction; SVV: stroke volume variation; PVV: pulse pressure variation; IVC: inferior vena cava; PaO_2_: arterial oxygen partial pressure; FiO_2_: inspired oxygen fraction; PaCO_2_: arterial carbon dioxide partial pressure. Time 0: after induction of general anaesthesia, before starting epidural analgesia. Time 1: 30 min after beginning epidural analgesia. Time 2: end of CRS, before abdominal filling. Time 3: end of HIPEC, before abdominal emptying. Time 4: end of procedure, after abdominal emptying and removal of abdominal drainage.

**Table 2:** Time course of biological data.

Time	Preoperative	ICU arrival	24 hours postoperative	48 hours postoperative	72 hours postoperative	*P* value
ASAT (UI/l)	22 (19–29)	147^∗^ (75–299)	153^∗^ (62–353)	142^∗^ (56–180)	94^∗^ (51–130)	<0.001
ALAT (UI/l)	19 (13–25)	94^∗^ (26–193)	117^∗^ (28–281)	105^∗^ (42–193)	89^∗^ (41–172)	<0.001
Serum lactate (mmol/l)	0.9 (0.7–1.2)	1.7^∗^ (1.3–3.5)	1.4 (1.2–2.3)	0.7 (0.5–1.7)	1.1 (0.7–1.2)	0.013
Serum albumin (g/l)	33 (26–36)	23^∗^ (19–27)	21^∗^ (17–24)	22^∗^ (21–24)	22^∗^ (20–24)	0.031
Serum creatinine (*μ*mol/l)	58 (55–79)	53^∗^ (44–70)	59 (50–78)	58 (48–74)	58 (52–65)	0.032

Data are expressed as median (quartile 25–quartile 75). *P* value results from a Friedman's two-way analysis of variance by rank. ∗ indicates a significantly different value compared to baseline with the Wilcoxon signed-rank test. ICU: Intensive Care Unit; ASAT: aspartate aminotransferase; ALAT: alanine aminotransferase.
